# Conventional Versus Videoscopic Inguinal Lymphadenectomy in Surgical Oncology: A Retrospective Observational Cohort Study

**DOI:** 10.1245/s10434-025-19041-7

**Published:** 2026-01-27

**Authors:** Nick Servaas, Loeki Aldenhoven, Merel A. Spiekerman van Weezelenburg, Elisabeth R. M. van Haaren, Alfred Janssen, Berry Meesters, Yvonne L. J. Vissers, Geerard L. Beets, James van Bastelaar

**Affiliations:** 1 Department of Surgery, Zuyderland Medical Centre, Sittard-Geleen, the Netherlands; 2 Department of Surgery, Maastricht University Medical Centre, Maastricht, the Netherlands; 3 GROW School for Oncology and Developmental Biology, Maastricht University, Maastricht, the Netherlands

**Keywords:** Inguinal lymphadenectomy, Groin dissection, Metastasis, Lymph node, Complications, Seroma, Melanoma, Rectum carcinoma

## Abstract

**Introduction:**

Inguinal lymphadenectomy is an important procedure in the treatment of various stage III malignancies and is notorious for postoperative complications, such as surgical site infections, seroma formation, skin flap necrosis, and wound breakdown. This study aims to investigate complication rates after conventional inguinal lymphadenectomy (CIL) and videoscopic inguinal lymphadenectomy (VIL) and to identify potential solutions to decrease complication rates.

**Methods:**

This was a single-center retrospective cohort study comparing CIL with VIL. Primary outcomes were the incidence of surgical complications, severity of surgical complications, readmissions, and surgical reinterventions. Additionally, drain use, flap fixation, antibiotics, unplanned visits, and oncological outcome were compared.

**Results:**

Between 2010 and 2024, 30 patients underwent CIL and 25 patients underwent VIL. The median operating time for CIL was 100 minutes (interquartile range [IQR] 88–119) compared with 120 minutes (IQR 105–139) for VIL (*p* = 0.017). The median duration of follow-up for patients in this cohort was 2.2 years (IQR 0.7–5.1). A total of 25 patients (83%) in the CIL group had at least one wound complication, compared with 13 (54%) in the VIL group (*p* = 0.020). Reinterventions in the first 30 postoperative days were performed in 12 patients (40%) after CIL, compared with none (0%) after VIL (*p* < 0.001). Disease-specific survival was not significantly different between procedures (*p* = 0.940).

**Conclusions:**

VIL reduced the rate of wound complications, severity of complications, and early surgical reinterventions compared with CIL but prolonged surgery time. The videoscopic procedure seems superior and offers a transformative approach that can be incorporated into contemporary surgical practice.

Inguinal lymphadenectomy (IL) has traditionally been regarded as a cornerstone surgical procedure in the treatment of stage III malignancies such as melanoma, Merkel cell carcinoma, penile cancer, vulvar cancer, and anorectal malignancies. Although the number of ILs for patients with melanoma reduced significantly after the publication of the Multicenter Selective Lymphadenectomy Trials I and II, IL is still routinely used in the treatment of patients with macroscopic stage III disease.^1–4^

IL is notorious for its complications, with complication rates of up to 77% reported in the literature.^5–7^ The most common complications are surgical site infections (SSI), seroma formation, and skin flap necrosis resulting in wound breakdown. Another common long-term complication is lymphedema, which occurs in up to 85% of patients.^5–7^ Videoscopic IL (VIL) and robot-assisted VIL (RAVIL) were introduced over the last decades with the aim to reduce complications.^8,9^ First introduced by Bishoff et al.^9^ for penile cancer, the feasibility of the videoscopic technique was later also reported for other tumors.^10–12^ In melanoma, the regional control was similar to that with the open approach.^13^ Complication rates were lower after videoscopic procedures than after conventional (open) IL (CIL).^14–17^ Moreover, the VIL procedure has a short learning curve and was consequently implemented as standard care.^18,19^

Although VIL reduced complication rates significantly, the incidence of wound and seroma-related complications after IL remains clinically challenging, with reported complication rates between 13 and 60%.^2,11–13^ The most common complication is seroma formation, with an incidence of 30–50%. Seroma formation is associated with SSI, wound breakdown, and skin flap necrosis.^2,13–15,20^ A definitive solution for preventing seroma formation after VIL has not yet been identified.^20^

This retrospective study aimed to investigate and compare complication rates after CIL and VIL and possibly identify solutions for reducing complication rates.

## Methods

### Study Design and Setting

This study was a single-center retrospective cohort study initiated in 2024. The study was performed in the department of surgical oncology at Zuyderland Medical Centre, Sittard-Geleen and Heerlen, the Netherlands. All data were retrospectively collected from electronic patient records available from 2010 onwards. Before the study started, the hospital’s medical ethical committee granted approval (Medical Ethical Review Committee Zuyderland; ID: METCZ2024012, approval date 22 January 2024), and the need for patient informed consent was waived. This report was written in compliance with the STROBE guidelines for cohort studies.^21^

### Study Population

All consecutive patients who underwent IL in Zuyderland Medical Centre between January 2010 and September 2024, who were aged ≥18 years, and who had completed at least 30-day follow-up were eligible for inclusion. Patients were divided into two cohorts: (1) CIL and (2) VIL, also known as minimally invasive inguinal lymph node dissection. VIL was first introduced in Zuyderland Medical Centre in 2016.

IL was performed as a completion lymphadenectomy after a positive sentinel node (prior to 2017) or as a total lymphadenectomy due to macroscopic stage III disease. IL can be performed as a (combined) primary procedure due to macroscopic stage III disease at baseline or as a secondary procedure due to either disease progression or recurrence in oncological follow-up. In certain cases, an iliac and obturator lymph node dissection was performed in the same procedure as IL. The treating surgeon chose the technique used for each patient. Although VIL was incorporated over time as surgical practice evolved, the surgeon could prefer CIL according to the extent of tumor involvement or ulceration of the skin in the groin area.

### Surgical Technique

#### Dissection Plane for Inguinal Lymphadenectomy

IL is performed by dissecting lymph nodes below Scarpa’s fascia and over the femoral vessels (ventral of fascia lata). The cranial border of the femoral triangle is Poupart’s ligament, the lateral border is formed by the sartorius muscle, and the medial border is formed by the adductor muscles. The field of dissection extends several centimeters cephalad to Poupart’s ligament.

#### Conventional (Open) Inguinal Lymphadenectomy

For CIL, a Lazy S incision is made in the inguinal skin fold, followed by dissection until Scarpa’s fascia. Skin flaps are created, and IL is performed, during which the saphenous vein is tied down at the Bishop’s Crook. The specimen is removed and marked for histopathological examination. Routinely, a low vacuum drain is placed in the dead space. Sartorius transposition to cover femoral vessels and flap fixation can be performed according to the surgeon’s preference, after which the subcutaneous layers and skin are closed.

#### Videoscopic Inguinal Lymphadenectomy

**Fig. 1:**
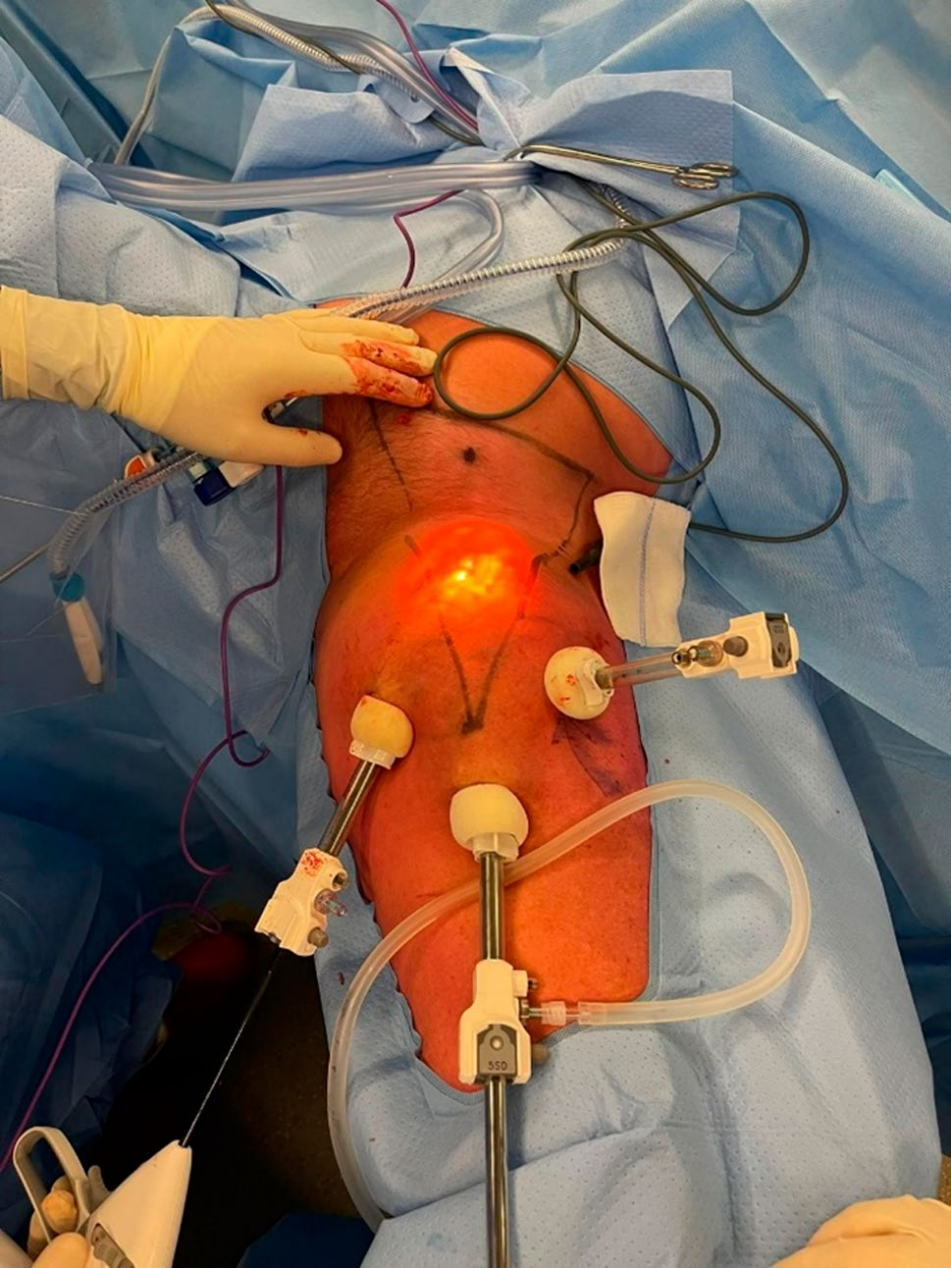
Right-sided videoscopic inguinal lymphadenectomy. The femoral triangle is drawn before surgery starts. Care is taken to avoid trocar placement above the dead space (femoral triangle), which remains after surgery

For VIL, the ‘landmarks’ of the femoral triangle are marked on the skin: spina iliaca anterior superior as craniolateral corner, tuberculum pubicum as craniomedial corner, and cross of the m. sartorius and m. adductor magnus as caudal ‘tip’ of the femoral triangle. The incision of the optical trocar is made 2–3 cm distal of the caudal tip of the femoral triangle. In addition, two incisions are made on the left and right for instrumental trocars, as can be seen in Fig. 1. Care is taken to avoid placing incisions in the skin flaps above the femoral triangle to prevent wounds above the postoperative dead space. The subcutaneous space is insufflated until 25 mmHg and lowered to 15 mmHg after 15 minutes of surgery. IL is performed, during which a Weck® Hem-o-lok® Polymer Ligation System (Teleflex Incorporated, Wayne, PA, USA) is used to seal the saphenous vein at the Bishop’s Crook. The specimen is removed and marked for histopathological examination. Routinely, a low vacuum drain is placed in the dead space. Flap fixation can be performed according to the surgeon’s preference, after which the subcutaneous layers and skin are closed.

### Postoperative Care and Follow-Up

Drains were removed by volume-controlled or time-controlled protocol after consulting the treating surgeon. During the timeframe of this study, the duration of postoperative drainage was empirically prolonged to at least 14 days because of the number of postoperative seromas and wound complications. Patients were seen by surgeons or wound care specialists in the outpatient clinic 2 weeks, 6 weeks, 3 months, and 6 months after surgery. When patients had fully recovered and all complications were resolved, they entered regular oncological follow-up protocols according to tumor type.

### Data Collection and Outcome Measures

Data from all ILs performed in Zuyderland Medical Centre between January 2010 and September 2024 were extracted from electronic patient records. A second researcher verified the validity and completeness of the extracted data using random case selection. Extracted variables included baseline characteristics, oncological characteristics, procedural characteristics, histopathological characteristics, postoperative complications, follow-up details, and survival data.

Primary outcomes were the incidence of surgical complications, severity of surgical complications, readmissions, and surgical reinterventions. Complications were defined as noted in Appendix 1, and severity was graded using Clavien–Dindo classification for surgical complications.^22^ Reinterventions were defined as surgical reinterventions in the operating room. This included reinterventions for bleeding complications, surgical debridement in the case of wound breakdown or necrosis, and surgical drainage of an abscess or infected seroma. Secondary outcomes were duration of drainage, use of flap fixation or tissue sealant, prophylactic use of antibiotics, unplanned visits, oncological outcome, and survival. Follow-up data for all patients were collected until the most recent visit.

### Data Analysis

The baseline characteristics, perioperative characteristics, and outcome measurements are described in detail. Continuous variables are described as mean ± standard deviation or, in case of severe skewness, as median (interquartile range). Categorical variables were reported as absolute numbers and percentages. Differences in baseline characteristics were assessed using the independent sample t-test or the Mann–Whitney U test for continuous data, depending on the skewness of the data. Group comparison for ordinal data was performed using the Mann–Whitney U test. Categorical data were tested using the Chi-squared test or Fisher’s exact test, depending on expected cell count. Trends in type of procedure, neoadjuvant therapy, and postoperative drain use were analyzed in depth to assess their potential risk for bias.

Differences in primary outcome measures and reinterventions were assessed by comparing patients with and without complications. Statistical testing was performed using univariable logistic regression, determining the relation between patient characteristics or surgical variables and outcome measure. Additionally, a multivariable logistic regression was performed with correction for potential confounders. Kaplan–Meier survival analysis was performed, and survival was compared by log-rank test. *P *values < 0.05 were considered statistically significant.

## Results

### Patients’ Characteristics

Between January 2010 and September 2024, a total of 55 patients underwent IL: 30 (55%) underwent CIL and 25 (45%) underwent VIL. All baseline characteristics are reported in Table 1. Of the 55 patients, 48 (87%) underwent IL due to skin cancer: 37 (67%) melanoma, five (9.1%) Merkel cell carcinoma, five (9.1%) squamous cell carcinoma, and one (1.8%) extramammary M. Paget. Five patients (9.1%) underwent IL due to metastatic rectal carcinoma. Patients undergoing VIL had a higher median Charlson Comorbidity Index score of 7 (IQR 5.5–9); those undergoing CIL had a median score of 5 (IQR 4–7.5) (*p* = 0.019). In total, 13 patients were treated with adjuvant systemic therapy: one (3.3%) in the CIL group and 12 (48%) in the VIL group (*p* < 0.001). A total of 12 patients (48%) in the VIL group underwent IL as a primary procedure due to macroscopic nodal disease at baseline compared with four patients (13%) in the CIL group (*p* = 0.023). Baseline characteristics showed no other statistical differences between groups.

**Table 1:** Baseline patient characteristics

Characteristic	Full cohort (*N* = 52)	CIL (*N* = 30)	VIL (*N* = 22)	*p* Value
Age, years	65.4 ± 11.4	65.2 ± 10.7	65.6 ± 12.4	0.909
*Sex*				0.657
Male	29 (53)	15 (50)	14 (56)	
Female	26 (47)	15 (50)	11 (44)	
BMI, kg/m^2^	26.2 (24.1–29.8)	29.2 (25.1–30.8)	25.8 (23.5–27.0)	0.051
*Active smoking*				0.766
Yes	12 (22)	7 (23)	5 (20)	
No	43 (78)	23 (77)	20 (80)	
*ASA score*				0.136
1	5 (9.1)	5 (17)	0 (0)	
2	35 (64)	18 (60)	17 (68)	
3	15 (27)	7 (23)	8 (32)	
Charlson comorbidity index	6 (4–9)	5 (4–7.5)	7 (5.5–9.0)	0.019*
Anticoagulant use	20 (39)	10 (33)	10 (46)	0.375
Systemic therapy before IL^a^	11 (21)	4 (13)	7 (32)	0.169
Adjuvant radiotherapy	13 (24)	6 (20)	7 (28)	0.487
Adjuvant systemic therapy	13 (24)	1 (3.3)	12 (48)	< 0.001*
*Timing of surgery*				0.005*
Primary	16 (29)	4 (13)	12 (48)	
Secondary	39 (71)	26 (87)	13 (52)	
Years between primary surgery and IL^b^	0.6 (0.1–2.3)	0.2 (0.1–1.6)	1.1 (0.5–2.6)	0.112
*Type of malignancy*				NA
Rectal carcinoma	5 (9.1)	1 (3.3)	4 (16)	
Melanoma	37 (67)	22 (73)	15 (60)	
Merkel cell carcinoma	5 (9.1)	2 (6.7)	3 (12)	
Squamous cell carcinoma	5 (9.1)	3 (10)	2 (8.0)	
M. Paget	1 (1.8)	1 (3.3)	0 (0)	
Mixed Mullerian tumor	1 (1.8)	0 (0)	1 (4.0)	
Undifferentiated carcinoma	1 (1.8)	1 (3.3)	0 (0)	

Data are presented as mean ± standard deviation, median (interquartile range), or n (%) unless otherwise indicated

*ASA* American Society of Anesthesiologists, *BMI* body mass index, *CIL* conventional (open) IL, *IL* inguinal lymphadenectomy, *IQR* interquartile range, *NA* not applicable, *SD* standard deviation, *VIL* videoscopic IL

^a^Patients receiving either adjuvant therapy after primary surgery (e.g. re-excision with sentinel node) < 1 year prior to a secondary IL, or patients receiving neoadjuvant treatment before IL

^b^Value was calculated for patients who received (different) primary surgery and underwent IL as a secondary intervention due to either recurrence or disease progression. This analysis included 26 patients from the open group, and 13 patients from the videoscopic group

### Perioperative Characteristics

Perioperative characteristics are reported in Table 2. IL was combined with (deep) iliac and obturator lymphadenectomy in 12 procedures (22%), of which 11 were performed with an open approach and one with a laparoscopic transperitoneal approach. Median operating time was 100 minutes (IQR 88–119) for CIL and 120 minutes (IQR 105–139) for VIL (*p* = 0.017). No statistically significant difference in total number of resected lymph nodes (IL + sentinel node) was found; the median was 11 nodes (IQR 7–15) for CIL and nine nodes for VIL (IQR 6–12) (*p* = 0.096). Flap fixation was used in 66% of the procedures. The use of flap fixation was comparable between the CIL and VIL groups (*p* = 0.717). Sartorius transposition was used in 28 patients (93%) undergoing CIL and in none of the patients during VIL procedures (*p* < 0.001)*.* Antibiotics were prophylactically prolonged for nine patients (30%) undergoing CIL and two patients (8.0%) undergoing VIL (*p* = 0.042).

**Table 2:** Perioperative characteristics

Characteristics	Full cohort (*N* = 55)	CIL (*N* = 30)	VIL (*N* = 25)	*p* Value
Type of surgery				0.340
IL	43 (78)	22 (73)	21 (84)	
Combined with deep IL	12 (22)	8 (27)	4 (16)	
Operating time, min^a^	114 (93–132)	100 (88–119)	120 (105–139)	0.017*
SN procedure before IL	26 (48)	15 (52)	11 (44)	0.571
Number of SNs resected from ipsilateral inguinal cavity before IL^b^	1 (1–2)	1 (1–2)	1 (1–2)	0.799
LNs resected (n)^c^	9 (6–12)	10 (7–12)	7 (6–11)	0.250
LNs resected, including SNs (n)^c^	10 (7–13)	11 (7–15)	9 (6–12)	0.092
Conversion	0 (0)	NA	0 (0)	NA
Sartorius transposition	28 (52)	28 (93)	0 (0)	< 0.001*
Flap fixation used	36 (66)	19 (63)	17 (68)	0.717
Postoperative antibiotics	11 (20)	9 (30)	2 (8.0)	0.042*

Data are presented as n (%) or median (interquartile range) unless otherwise indicated

*CIL* conventional (open) IL; *IL* inguinal lymphadenectomy, *IQR* interquartile range, *LN* lymph node, *NA* not applicable, *SN* sentinel node, *VIL* videoscopic IL

^a^Value was calculated for procedures that were not combined with other procedure; only superficial IL was performed in these patients. This analysis included 16 patients in the open group, and 15 patients in the videoscopic group

^b^Value was calculated for patients who underwent sentinel node procedure prior to IL. Only the number of nodes removed from the ipsilateral inguinal cavity were included in this analysis

^c^Three patients had conglomerate LNs in the dissection specimen: two in the CIL group and one in the VIL group

### Trends of Care in Our Institution

**Fig. 2:**
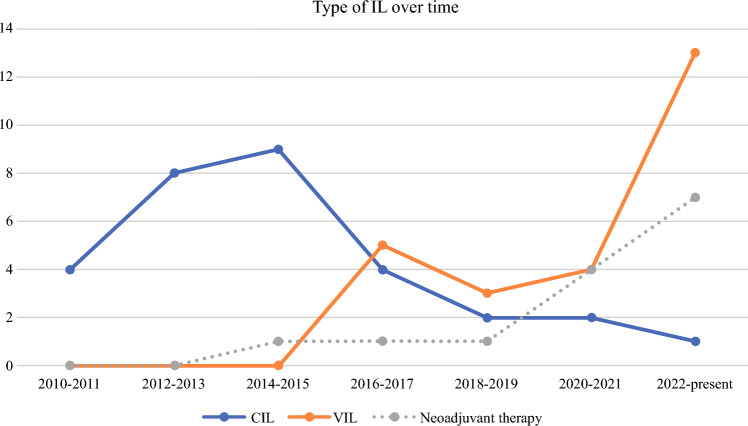
Number of conventional inguinal lymphadenectomy (CIL) and videoscopic inguinal lymphadenectomies (VILs) per given period of time, with trend in neoadjuvant treatment before inguinal lymphadenectomy

VIL was introduced in Zuyderland Medical Centre in 2016 and became the standard of care over time. The number of VIL procedures gradually increased, whereas CIL procedures reduced, and the use of neoadjuvant systemic therapy also increased (Fig. 2).

**Fig. 3:**
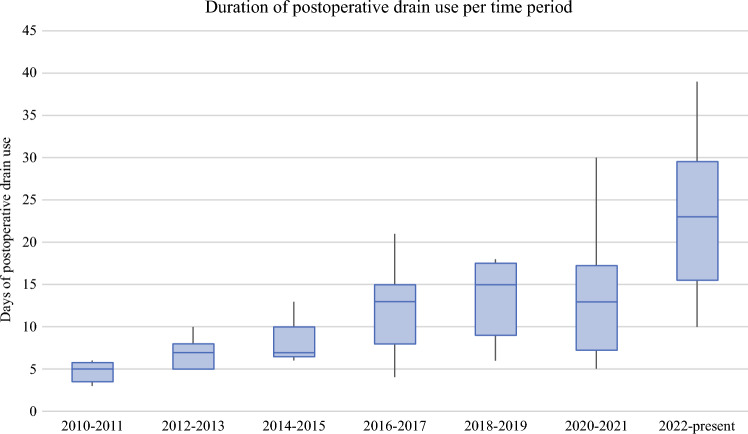
Boxplots of median days of postoperative drain use after inguinal lymphadenectomy per time. Error bars mark the range of lowest and highest number of days until drain removal

The third time trend was an increased duration of postoperative low vacuum drainage in an attempt to decrease the number of wound complications. The overall median duration of drainage was 7 days (IQR 5–10.5) after CIL and 16 days (IQR 14–26) after VIL (*p* < 0.001). As shown in Fig. 3, this increased from 5 days in 2010 to 23 days at the time of writing. The duration of postoperative drainage had no effect on the incidence of wound complications and its severity.

### Outcome Per Procedure

Table 3 shows the primary and secondary outcome measures per procedure. VIL was associated with a significant reduction in the severity of complications compared with CIL (*p* = 0.017). One patient in the VIL group died postoperatively due to a complication associated with laparoscopic ilio-obturator lymphadenectomy. The patient experienced an incarcerated herniation of the small bowel through an iatrogenic defect in the peritoneal lining, causing necrosis, perforation, and severe sepsis. This patient was censored in further analyses as this complication was considered not associated with the superficial IL.

**Table 3:** Primary and secondary outcome measures

Characteristic	CIL (*N* = 30)	VIL (*N* = 25)	*p* value
*Clavien–Dindo grade of most severe complication*			0.017*
No complication	2 (6.7)	6 (24)	
Grade I	2 (6.7)	3 (12)	
Grade II	13 (43)	10 (40)	
Grade IIIa	0 (0)	1 (4.0)	
Grade IIIb	13 (43)	4 (16)	
Grade IV	0 (0)	0 (0)	
Grade V^a^	0 (0)	1 (4.0)	
	**CIL (N = 30)**	**VIL (N = 24)**	
Wound complications^b^	25 (83)	13 (54)	0.020*
*Type of wound complication*			
Hematoma	1 (3.3)	0 (0)	
Bleeding	1 (3.3)	0 (0)	
Seroma with wound breakdown	0 (0)	2 (8.3)	
Infected seroma	2 (6.7)	6 (25)	
SSI	7 (23)	2 (8.3)	
Necrosis^c^	14 (47)	3 (13)	
*Amount of wound complications*^*b*^			0.058
No complication	4 (13)	12 (52)	
One complication	13 (43)	5 (22)	
Multiple complications	13 (43)	6 (26)	
Seroma	23 (77)	22 (92)	0.270
Clinically significant seroma	17 (57)	17 (71)	0.284
Patients receiving at least one seroma aspiration	9 (30)	11 (46)	0.231
Amount of seroma aspirations	1 (1–2)	2 (1–4.5)	0.283
Lymphedema	22 (73)	16 (67)	0.594
Readmission < 30 days	11 (37)	5 (21)	0.205
Readmission during follow-up	12 (40)	11 (46)	0.667
Reintervention < 30 days	12 (40)	0 (0)	< 0.001*
Reintervention during follow-up	13 (43)	6 (25)	0.161
*Reason for reintervention during follow-up*			
Bleeding	1 (3.3)	0 (0)	
Necrosis^c^	11 (37)	4 (16)	
Abscess	1 (3.3)	0 (0)	
Infected seroma	0 (0)	2 (8.0)	
Days until reintervention	13 (11.5–20)	59 (41.5–76)	0.001*

Data are presented as n (%) or median (interquartile range) unless otherwise indicated

*CIL* conventional inguinal lymphadenectomy, *SSI* surgical site infection, *VIL* videoscopic inguinal lymphadenectomy

^a^Grade V complication was *NOT* associated with inguinal lymphadenectomy but was associated with a laparoscopic transperitoneal ilio-obturator lymphadenectomy in the same session. Patient was censored in further analyses into complications as this complication is associated with a different procedure

^b^Seroma formation was only registered as a wound complication in case of secondary consequences affecting wound healing (e.g. seroma leading to wound dehiscence or wound necrosis, or infected seroma)

^c^Two cases of necrosis due to in-basin tumor recurrence were observed in this cohort: one patient in the CIL group and one patient in the VIL group. In both cases a surgical reintervention was required

A total of 38 patients (70%) had at least one wound complication: 25 patients (83%) in the CIL group and 13 patients (54%) in the VIL group (*p* = 0.020). Multiple complications occurred in 13 patients in the CIL group and six patients in the VIL group (*p* = 0.058). The incidence of SSIs and skin flap necrosis was higher in the CIL cohort, whereas the VIL cohort had a higher incidence of seromas resulting in either wound breakdown or infection.

Seroma, clinically significant seroma (CSS), lymphedema, and readmissions were not significantly different across groups. Reinterventions in the first 30 postoperative days were performed in 12 patients (40%) after CIL and no patients (0%) after VIL (*p* < 0.001). Late reinterventions were performed in seven patients (13%): one (3.3%) in the CIL group and six (25%) in the VIL group. No significant difference in the need for surgical reinterventions during follow-up were found (*p* = 0.161). Reinterventions were performed after a median of 13 days (IQR 11.5–20) in the CIL group, significantly earlier than the median 59 days (IQR 41.5–76) in the VIL group (*p* = 0.001).

### Characteristics Contributing to Complications

Multiple univariable and multivariable regression analyses were performed within the cohort to assess the effect of baseline characteristics (sex, age, body mass index [BMI], smoking, American Society of Anesthesiologists score, systemic treatment), perioperative characteristics (CIL or VIL, operation time, use of flap fixation), and postoperative characteristics (prolonged use of antibiotics, days of drainage) on seroma, CSS, lymphedema, and wound complications.

No baseline characteristics, perioperative measures, or postoperative measures influenced the occurrence of seroma, CSS, or lymphedema after IL. The use of VIL was associated with a significant reduction in the occurrence of wound complications compared with CIL (odds ratio [OR] 0.236 [95% CI 0.068–0.826], *p* = 0.024). An increase in BMI was associated with a clinically relevant increased risk for the occurrence of wound complications, although this was not statistically significant (OR 1.171 [95% CI 0.989–1.385], *p* = 0.067). A binomial logistic regression was performed to ascertain the effect of VIL and BMI on the likelihood of wound complications. The model provided an area under the receiving operator curve of 0.744. After correction for confounders, only the type of dissection remained statistically significant (*p* = 0.046). Patients undergoing CIL had 3.7 times the odds of exhibiting a wound complication compared with patients undergoing VIL (95% CI 1.02–13.51).

### Follow-up and Oncological Outcome

The median duration of follow-up for patients in this cohort was 2.2 years (IQR 0.7–5.1). Patients had a median of five unplanned visits (IQR 2–8) (e.g. due to complications) before being transferred to regular oncological follow-up. Of the 55 patients, 11 (20%) completed oncological follow-up, 20 (36%) remain in oncological follow-up at the time of writing, two (3.6%) were lost to follow-up, and 22 (40%) died during follow-up. In-basin recurrence was seen in five patients (9.1%) during follow-up: two (6.7%) in the CIL group and three (12%) in the VIL group (*p* = 0.650). This led to two surgical reinterventions (one patient in each group; *p* = 1.000). In both cases, the tumor recurrence led to necrosis, for which wound debridement was performed. No additional lymph node extractions within the same nodal basin were performed. In total, 19 patients (35%) died due to disease recurrence or progression, one patient (1.8%) died due to surgical complications, and two patients (7.3%) died of other causes.

**Fig. 4:**
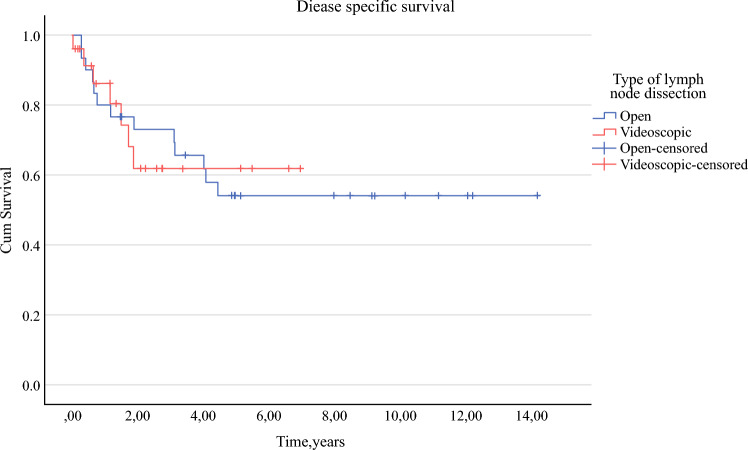
Kaplan–Meier estimates of disease-specific survival (death due to disease progression/recurrence or surgical complications) per procedure. Censoring was plotted for the duration of the follow-up

Follow-up duration was significantly shorter for VIL (1.4 years [IQR 0.5–2.7]) than for CIL (4.2 years, IQR 1.1–8.1) (*p* = 0.011). Disease-specific survival for CIL and VIL are shown in Figure 4. No significant differences in disease-specific survival between procedures were found (*p* = 0.940).

## Discussion

In this single-center retrospective cohort study, CIL and VIL procedures were compared among 55 consecutive patients from 2010 to 2024.

### Technical Considerations

VIL was introduced in our institution as a potential solution to reduce the complication rates and morbidity associated with CIL. VIL required a longer operation time, which corresponds with findings in the literature.^17,23^ VIL is more technically challenging than CIL, and additional time is required until lymphadenectomy is performed (due to trocar placement and additional tissue dissection towards the femoral triangle), which explains the increased operation time. The learning curve for VIL is short and does not significantly increase the time in surgery.^18,19^ The amount of lymph nodes harvested showed no significant difference across both procedures in this cohort. A recent meta-analysis by Patel et al.^17^ reported a greater amount of harvested lymph nodes for CIL than for VIL. VIL reduces the direct exposure to tissue and may therefore lead to fewer harvested lymph nodes. Studies have shown that the oncological outcome of both procedures is comparable.^14–17^ The reduced number of resected lymph nodes does not compromise oncological safety in the literature; however, in our cohort, there was no difference in numbers of harvested lymph nodes. VIL is considered a safe oncological alternative to CIL when performed by a trained surgeon.^14–17^ Disease-specific survival did not differ between the groups in this cohort. Notably, a higher proportion of patients received neoadjuvant treatment in the VIL group. Subsequently, a statistically significant increase in adjuvant systemic treatment was observed for the VIL group. This increase in systemic treatment may have influenced the oncological outcome, so survival outcomes related to the procedure should be interpreted with caution.

### Complications

The introduction of VIL led to a significant reduction in wound complications, Clavien–Dindo grade III complications, and early reinterventions in our institution. Wound complications occurred in 54% of patients after VIL and 83% after CIL. Approximately half of the patients with a wound complication in the CIL group had to undergo a surgical reintervention. Skin flap necrosis caused most of these reinterventions, and no early reinterventions were necessary in the VIL group. The occurrence of wound complications in the CIL group had an OR of 3.7 when compared with VIL. VIL reduces both the number and the severity of complications while simultaneously reducing the need for surgical reinterventions. Among patients with complications in the CIL group, 56% developed skin flap necrosis, making it the most common complication. IL is notorious for postoperative seromas, with an incidence up to 77%.^5–7^ In VIL, care is taken to avoid surgical incisions directly above the femoral triangle. In CIL, the surgical incision is placed directly above the dead space. As a consequence, seroma formation exerts severe pressure on the wound bed, often resulting in skin flap necrosis and wound breakdown. The different anatomical position of the surgical incisions and the extent thereof in relation to the dead space is considered to play a crucial role in the reduction of surgical complications between the two procedures.^14–17^

The reduction of surgical reinterventions is related to the type and timing of the primary wound complication. Surgical debridement is often necessary in cases with skin flap necrosis (most common in the CIL group), whereas seroma aspiration and administration of (intravenous) antibiotics may suffice for infected seroma (most common in the VIL group). Drainage time was prolonged in our institution over the years, whereas VIL became the standard of care. The time to drain removal was significantly higher in the VIL group. This may bias the reduction in postoperative complications, especially CSS and related complications such as skin flap necrosis, since continuous drainage of seroma reduces stress on surgical wounds, leading to potential overestimation of the protective effect of VIL.

### Drainage Time and Seroma Preventive Measures

Preventive measures to reduce postoperative seromas and CSS were extensively analyzed in this cohort. Protocols and time to drain removal altered during the timeframe of this study, so the effect of drainage could not be properly compared between procedures. The occurrence of seroma and CSS was not influenced by time to drain removal in this cohort. Pouwer et al.^24^ showed that volume-controlled drainage (drain removed when drain output was < 30 ml/24 h) reduced the number of wound complications when compared with short drainage (< 5 days) after CIL. Complete drain removal using volume-controlled protocols is non-inferior to progressive drain removal after CIL.^25^ Several studies have identified that RAVIL leads to shorter drainage than does CIL when using volume-controlled removal protocols.^26,27^ Although complete volume-controlled drain removal seems feasible after CIL, efficacy after VIL has not been determined. No optimal window for drain removal after IL has yet been established. It should be noted that volume-controlled drain removal after IL can be problematic because of persistent high lymphatic drainage when patients mobilize postoperatively. Moreover, drain removal thresholds are arbitrary and could unnecessarily prolong drain dependence.

The use of flap fixation and tissue sealants had no effect on seroma formation or CSS in this cohort. The use of tissue sealants after IL has been previously studied, and meta-analysis by Gerken et al.^28^ showed no reduction in either postoperative seroma or CSS. However, a more recent randomized controlled trial showed promising results for the use of fibrin glue to reduce postoperative seromas.^29^ The use of tissue sealants after IL remains a subject of debate and should be evaluated in a larger cohort. The routine use of postoperative antibiotics did not improve outcome measures, which corresponds with findings in the literature.^30,31^

### Strengths and Limitations

This study was conducted in a high-volume center for oncology in the Netherlands. Regular follow-up consultations were conducted by surgeons or wound care specialists for at least 6 months after surgery or until all complications had resolved. The frequent follow-up meant that wound complications were assessed regularly, resulting in valid and accurate measurement of defined primary and secondary outcome measures. Length of follow-up ranged from 2 months up to 14 years, further strengthening the validity of our results. Findings in this study are mainly limited by sample size, as only 55 patients were included over 14 years. Another limitation was the retrospective design of this study, leading to limitations in, for example, registration of drain output. Trends in care (e.g. increasing use of (neo)adjuvant therapy or empirically prolonged drainage time) may lead to bias in this cohort.

### Recommendations for Current Practice and Future Research

Based on the results of this study and findings in the literature, we strongly advise that IL should be performed using a minimally invasive approach, such as VIL or RAVIL. VIL leads to a significant reduction in wound complications, severity of complications, and surgical reinterventions. The learning curve is short and should not be a barrier to implementation. The incidence of seroma formation after IL remains high, and potential solutions have not yet been identified. No optimal window for drain removal after IL has yet been established. Complete drain removal using a volume-controlled protocol is feasible after CIL and should be assessed after VIL. The effect of flap fixation and the use of tissue sealants remains highly debatable. Prolonged use of antibiotics after IL is redundant. Findings in the literature are often limited by sample size and the retrospective design of the studies, whereas meta-analyses have high heterogeneity and risk of bias.

## Conclusions

VIL reduced wound complication rates, the severity of complications, and the need for early surgical reinterventions when compared with CIL but prolonged surgery time. The videoscopic approach for IL seems to be superior to the conventional approach and offers a transformative approach that can be incorporated into contemporary surgical practice.

## Appendix 1: Definition of complications

**Table:**

Complication	Definition
Seroma	Collection of serous fluids in the dead space after surgery
Clinically significant seroma (CSS)	Seroma leading to secondary complications such as infection of seroma, impaired wound healing (e.g. necrosis or wound dehiscence) or pain, which require an intervention
Lymphedema	Localized swelling of the body caused by an abnormal accumulation of lymphatic fluids
Surgical site infection (SSI)	Localized infections that occur in or around the wound created by an invasive surgical procedure, in superficial layers of the skin
Deep wound infection	Wound infection spreading to deeper layers of the skin, e.g. cellulitis of erysipelas
Necrosis	Death of tissue caused by disease or injury
Dehiscence	Partial or total separation of previously approximated wound edges, due to a failure of proper wound healing
Abscess	An enclosed collection of pus in tissues, organs, or confined spaces in the body
Hematoma	An area of blood that collects outside of the larger blood vessels
Bleeding	Blood actively escaping from the circulatory system due to damaged blood vessels
Readmission	Readmission of a patient in the hospital within 30 days after primary surgery
Reintervention	Surgical reintervention under general anesthesia (Clavien–Dindo grade IIIB or higher) within 30 days after primary surgery
Mortality	In-hospital death or death within 30 days after primary surgery
